# Editorial: Network pharmacology and traditional medicine: Setting the new standards by combining *In silico* and experimental work

**DOI:** 10.3389/fphar.2022.1002537

**Published:** 2022-10-21

**Authors:** Xin Wang, Yuanjia Hu, Xuezhong Zhou, Shao Li

**Affiliations:** ^1^ MOE Key Laboratory of Bioinformatics, BNRIST/Department of Automation, Institute for TCM-X, Tsinghua University, Beijing, China; ^2^ State Key Laboratory of Quality Research in Chinese Medicine, Institute of Chinese Medical Sciences, University of Macau, Taipa, Macau SAR, China; ^3^ School of Computer and Information Technology, Beijing Jiaotong University, Beijing, China

**Keywords:** network pharmacology, traditional medicine, experimental work, network target, complex disease

## Network pharmacology: A breakthrough point for traditional medicine research in the era of biomedical big data and artificial intelligence

With the rise of the interdisciplinary Frontier such as system medicine and bioinformatics, a new generation of pharmaceutical research paradigm characterized by network and system has attracted more and more attention in the era of biomedical big data and artificial intelligence. Network pharmacology is a Frontier research field that systematically illustrates mechanisms of drugs and guides the research and development of drugs into clinical diagnosis and treatment. It is an original interdisciplinary subject of pharmacology, system biology, bioinformatics, network science, and other related disciplines. Network pharmacology emphasizes the overall perspective of the biological networks by analyzing the molecular association between drugs and their treatment objects. Traditional medicine (TM) has accumulated ample treatments from long-term clinical practice. It is worth noting that TM is characterized by holistic, personalized, multi-component, multi-target and multi-pathway therapy. To systematically reveal the biological basis of the overall diagnosis and treatment of TM, a key concept derived from the multi-target nature of traditional medicine has been proposed, termed as “network target”, shifting away from the current “single target” research paradigm (Li, 1999; Li et al., 2011; Li and Zhang, 2013). The network target refers to treating the biological network underlying diseases as a therapeutic target in order to decipher systematic mechanisms of action for multi-target drugs, particularly for traditional medicine. The concept, methodologies, and case studies related to network target were prior to the introduction of the network pharmacology term and laid the foundation for network pharmacology (Li et al., 2007). Afterwards, the new term “network pharmacology” was introduced (Hopkins, 2007). The original theory and methodologies of network target play a key role in the origin and development of network pharmacology and are widely used in TM research.

## Trends in network pharmacology: Combining *in silico*, experimental and clinical work

Network pharmacology tends to integrate computational, experimental techniques and clinical investigations, and create favorable conditions for exploring the pharmacological mechanism of TM (Wang X. et al., 2021). It intends to systematically understand and reveal the biological basis of complex diseases and drug effects. Emerging experimental technologies such as high-throughput screening, single-cell sequencing, and gene editing have promoted the development of network pharmacology (Guo et al., 2019; Zhang et al., 2019). It is noteworthy that the first international standard in the field of network pharmacology (“*Guidelines for Evaluation Methods of Network Pharmacology*”) was published in 2021 (Li, 2021b). This standard specifies the principles, strategies, and evaluation criteria for data Research Topic, network analysis, and experimental assessment in the process of network pharmacology research and is applicable to researchers engaged in network pharmacology study. It is helpful to promote the scientization and standardization of network pharmacology and TM research. At the same time, the first monograph (“*Network Pharmacology*”) was published in Springer Press (Li, 2021a). This book is devoted to systematically introducing the research progress of the theories, methods, and applications of network pharmacology.

As a new interdisciplinary field, TCM network pharmacology is facing some problems and challenges while developing rapidly, including 1) Different criteria make TCM-related databases difficult to compare and integrate. At the same time, there is homogenization in the screening of active ingredients; 2) The clinical effectiveness of TCM prescriptions is often ignored in network pharmacology studies; 3) The results of network pharmacological analysis need to be further verified by experimental evaluation. It is suggested to standardize from the following aspects. In the terms of data collection, it is necessary to integrate multiple sources and combine them with a variety of experimental methods to collect data for effective parts, single herbal extracts or herbal formulae. In terms of algorithms, the application scope, characteristics and limitations of different methods are understood to further enhance the verification of the results of network pharmacology analysis. Validation of network pharmacology analysis should be in accordance with best practice guidelines pharmacological studies on plant effective parts/herbal formulae/natural products (Heinrich et al., 2020). For example, researchers need to evaluate the overall pharmacological effects of single herbal extracts first, and then test the specific mechanism of action or targets.

**FIGURE 1:**
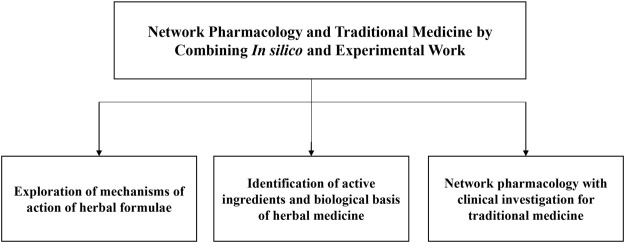
Summary of the 39 articles in this research topic.

With the new progress in network pharmacology and TM, Frontiers in Pharmacology organized a Research Topic entitled “Network Pharmacology and Traditional Medicine: Setting the New Standards by Combining *In silico* and Experimental Work” to present recent advances pertaining to network pharmacology and traditional medicine by combining computational and experimental techniques (Figure 1). This topic consists of 37 original research and two reviews and has attracted wide attention with over 101,000 views and more than 36,500 article downloads.

## Mechanisms of action of herbal formulae

TM prescriptions based on herbal formulae are widely used to prevent and treat diseases. Herbal formula is composed of various kinds of herbs with specific therapeutic effects. TM prescriptions have curative effects and fewer side effects in the treatment of diseases. However, TM prescriptions contain various ingredients, resulting in a problem of unclear targets and mechanisms. Network pharmacology provides a new strategy and powerful methodology for revealing the mechanisms of action of herbal formulae. Network pharmacology studies for herbal formulae first need to evaluate the potential effects of herbal formulae, and a reasonable and therapeutically relevant dose range should be tested. Exploring the mechanisms of action of herbal formulae requires the construction of appropriate and credible animal and cell models for experimental verification. Chemical composition and detection methods should be stated. After rigorous criterion and peer review, this research topic collected 22 original articles that focus on the mechanisms of action of herbal formulae on a variety of complex diseases.

In this Research Topic issue, a group of papers investigated the mechanisms underlying herbal formulae on metabolic diseases including diabetes and non-alcoholic fatty liver disease. Xu et al. utilized network pharmacology-based analysis, *in vivo* and *in vitro* experiments to explore the targets and antidiabetic effects of Gegen QinLian Decoction (GQL). They found that GQL affects blood glycemic levels, ameliorates inflammatory symptoms, and liver and pancreas tissue injury by regulating TNF and PI3K–AKT signaling pathways . Moreover, researchers unveiled that induction of autophagy may be the major mechanism for Kun-Dan Decoction to improve insulin resistance and metabolic syndrome (Su et al.). Wei et al. integrated network-based algorithms and experimental studies to indicate that Sinisan reduced hyperlipidemia, liver steatosis and inflammation by inhibiting JAK2/STAT3 signal. In addition, network pharmacology and experimental work elucidated the mechanisms of action of Hugan Tablets for drug-induced liver injury (Lv et al.) and Shenerjiangzhi formulation on hyperlipidemia (Zhang et al.).

Some herbal formulae mainly treat neurological or mental disorders. In this research topic issue, Qu et al. used network pharmacology to explore potential targets and depress-related pathways of Huang-Lian Jie-Du Decoction. The antidepressant activity, potential targets and active components were further evaluated by *in vivo* depression-associated models. In addition, Yan et al. uncovered the antidepressant mechanism of Xiaoyaosan in CUMS-induced depressed mouse model based on network pharmacology analysis and *in vivo* experimental assessments. Xiaoyaosan could alleviate targets (RIPK1, RIPK3, p-MLKL) in necroptosis and improve the hippocampal function and neuroinflammation in depressed mice. Another paper proposed an integrated computational and experimental approach to analyze the mechanisms of ingredients of Qingkailing injection targeting cerebral ischemic networks (Wang et al.).

Another group of articles focused on the mechanisms of action of herbal formulae for arthritis. Two papers integrating network computational analysis and experimental assessment to explore the mechanism of Bushen Zhuangjin Decoction on osteoarthritis by regulating NF-κB signaling pathway (Xu et al.) and identification of inhibiting TLR/MyD88/NF-κB signaling pathway by Duhuo Jisheng Decoction on Osteoarthritis (Liu et al.). Furthermore, by combining network pharmacology analysis and *in vivo* and *in vitro* experiments, a paper showed that Wutou Decoction attenuates rheumatoid arthritis *via* suppressing angiogenesis and regulating the PI3K/AKT/mTOR/HIF-1α pathway (Ba et al.).

## Identification of active ingredients and biological basis of herbal medicine

Traditional medicine often exerts therapeutic effects by affecting multiple targets and ingredients. Network pharmacology is to analyze the biological basis of herbs and their compounds by combining computational and experimental approaches and identifying the bioactive ingredients from numerous herbs for various diseases. Network pharmacology analysis for active ingredients should be based on physiological and pathological characteristics of dynamic network analysis to avoid homogenization of active ingredient screening. Active ingredients and multiple targets analyzed by high-throughput screening should be further validated by other accurate experiments. In addition, it is also necessary to clarify the specificity of the key drivers and targets of the diseases, and appropriately consider the weights in network pharmacology analysis. There are 13 papers published in this Research Topic to explore the active ingredients and biological basis of herbal medicine.

There are two papers illustrating the anti-tumor mechanisms and active ingredients of herbs by combining network pharmacology, pharmacokinetics analysis and biological experiments. Wang et al. determined that the active ingredient in *Scutellaria baicalensis* Georgi [Lamiaceae] related to the onset and development of gliomas was Wogonin by up-regulating pro-apoptotic factors and downregulating anti-apoptotic factors. Another paper showed that ten ingredients identified in the aqueous extract of *Oldenlandia hedyotidea* (DC.) Hand.-Mazz [Rubiaceae] have anti-cancer effects *in vitro* experiments of esophageal cancer (Zhao et al.).

Traditional herbs are commonly used for chronic diseases including chronic obstructive pulmonary disease (COPD) and chronic kidney disease. Critical ingredients and anti-inflammatory effects of five ingredients combination from herbal medicines used for COPD were explored by network pharmacology network analysis integrated with experimental validation. They found that five compounds treated COPD *via* the regulation of the underlying inflammatory process such as IL-17, innate immune response–activating signal transduction (Li et al.). Yong et al. identified the active ingredient luteolin and therapeutic mechanisms of *Perilla frutescens* (L.) Britton [Lamiaceae] in the treatment of chronic kidney disease through network-based computational analysis, pharmacokinetic analysis and *in vitro* experiments (Yong et al.).

Traditional medicine herbs are also used for treating liver injury and lung injury. To identify active ingredients of *Scutellaria baicalensis* Georgi [Lamiaceae], a paper extracted the volatile active substances by GC–MS and identified candidate targets for lung injury by network pharmacology approaches (Zhu et al.). They verified wogonin in the specific regulatory pathways of PI3K-Akt signaling and IL-17 signaling through molecular biological experiments. In another paper, Wu et al. analyzed the components of *Citri Reticulatae* Pericarpium (a dried and mature pericarp *Citrus × aurantium* L. [Rutaceae]) and validated potential molecular functions in the treatment of liver injury through network pharmacology and *in vitro* experiments (Wu et al.).

Moreover, UPLC-QTOF-MS/MS and network pharmacology approaches were utilized to identify the chemical spectrum of the dried root of *Aconitum carmichaeli* Debeaux [Ranunculaceae] (*Aconiti Radix* cocta, ARC) and potential targets and pathways (Ye et al.). They uncovered the underlying mechanism of ARC for gouty arthritis by inhibiting the inflammatory factors. The network pharmacology computational approach integrates experimental assessment for illustrating the pharmacological mechanism of the dried seeds of Ziziphus jujuba Mill. [Rhamnaceae] and bioactive ingredients in the treatment of insomnia (Bian et al.). In addition, active ingredients Diosgenin from the rhizome of *Dioscorea nipponica* Makino [Dioscoreaceae] was determined to ameliorate Grave’s disease *via* inhibiting the phosphorylation and activation of PI3K-AKT and Rap1-MEK signaling pathways by integrating network pharmacology and experimental validation (Xin et al.).

## Network pharmacology with the clinical investigation for traditional medicine

The research on network pharmacology and traditional medicine shows the development trends of the in-depth integration among computational approaches, clinical trials and experiments. Clinical trials are an important data source for network pharmacology research. The potential targets, herbal prescriptions, biological functions obtained through network pharmacology analysis need to be verified, and clinical trial is the most rigorous and convincing verification strategy. There are two reviews and one original research focused on network pharmacology approaches and clinical-based evidence of traditional medicine.

A paper compared the efficacy and safety of conventional treatments (CTs) to those that included traditional Chinese medicine injections in patients with combined coronary heart disease and heart failure (CHD-HF) by network meta-analysis. They found that traditional Chinese medicine injections combined with CTs are better than CTs alone in treating CHD-HF, and different injections improve different outcomes respectively (Wei et al.). Another paper explored the anti-rheumatoid arthritis mechanism of a clinical evidence-guided herbal medicine Shuji tablet by network pharmacology and *in vivo* experimental validation. Shuji tablet significantly alleviated rheumatoid arthritis of adjuvant-induced arthritis rats in the regulation of PI3K-Akt, IL-17, FoxO, Rap 1 and other signaling pathways (Dai et al.). In addition, Zhang et al. collected relevant clinical trials and analyzed experimental evidence, in which bioactive ingredients of *Salvia miltiorrhiza* Bunge [Lamiaceae] (Danshen) attenuated rodent colitis in the management of intestinal integrity, gut microflora, cell death, immune conditions, cytokines, and free radicals (Zhang et al.). The network pharmacology approach was applied to describe sophisticated mechanisms of Danshen in treating inflammatory bowel disease in a holistic view.

## Conclusion

In summary, the Research Topic of 39 articles contributed to this Research Topic demonstrates the booming attraction to network pharmacology and TM. Network pharmacology integrating experimental work is a key role to promote the innovation and development of TM. In this Research Topic, the articles cover broad applications of network pharmacology computational approaches combing biological experiments on TM including the illustration of pharmacological mechanisms of herbal formulae, identification of biological functions and targets of herbal active ingredients, and reviews on network pharmacology methodologies combining experimental and clinical evidence for TM treatment. These studies are regarded to provide support for clinical practice and the interpretation of mechanisms of traditional medicine by in-depth integration of network pharmacology of computational, experimental and interdisciplinary field. This Research Topic provides evidence and guidance for high-quality research in network pharmacology and traditional medicine, as well as encouraging advances in this novel and frontier research field.
